# Development, Validation, and Reliability of a Sedation Scale in Horses (EquiSed)

**DOI:** 10.3389/fvets.2021.611729

**Published:** 2021-02-16

**Authors:** Alice Rodrigues de Oliveira, Miguel Gozalo-Marcilla, Simone Katja Ringer, Stijn Schauvliege, Mariana Werneck Fonseca, Pedro Henrique Esteves Trindade, José Nicolau Prospero Puoli Filho, Stelio Pacca Loureiro Luna

**Affiliations:** ^1^Department of Veterinary Surgery and Animal Reproduction, School of Veterinary Medicine and Animal Science, São Paulo State University Universidade Estadual Paulista (UNESP), Botucatu, Brazil; ^2^The Royal (Dick) School of Veterinary Studies and The Roslin Institute, The University of Edinburgh, Edinburgh, United Kingdom; ^3^Section Anaesthesiology, Department of Clinical Diagnostics and Services, Vetsuisse Faculty, University of Zürich, Zurich, Switzerland; ^4^Department of Surgery and Anaesthesia of Domestic Animals, Faculty of Veterinary Medicine, Ghent University, Ghent, Belgium

**Keywords:** acepromazine maleate, horses, detomidine, scales, methadone, α-2 adrenergic receptor agonist

## Abstract

The lack of standardization of sedation scales in horses limits the reproducibility between different studies. This prospective, randomized, blinded, horizontal and controlled trial aimed to validate a scale for sedation in horses (EquiSed). Seven horses were treated with intravenous detomidine in low/high doses alone (DL 2.5 μg/kg + 6.25 μg/kg/h; DH 5 μg/kg +12.5 μg/kg/h) or associated with methadone (DLM and DHM, 0.2 mg/kg + 0.05 mg/kg/h) and with low (ACPL 0.02 mg/kg) or high (ACPH 0.09 mg/kg) doses of acepromazine alone. Horses were filmed at (i) baseline (ii) peak, (iii) intermediate, and (iv) end of sedation immediately before auditory, visual and pressure stimuli were applied and postural instability evaluated for another study. Videos were randomized and blindly evaluated by four evaluators in two phases with 1-month interval. Intra- and interobserver reliability of the sum of EquiSed (Intraclass correlation coefficient) ranged between 0.84–0.94 and 0.45–0.88, respectively. The criterion validity was endorsed by the high Spearman correlation between the EquiSed and visual analog (0.77), numerical rating (0.76), and simple descriptive scales (0.70), and average correlation with head height above the ground (HHAG) (−0.52). The Friedman test confirmed the EquiSed responsiveness over time. The principal component analysis showed that all items of the scale had a load factor ≥ 0.50. The item-total Spearman correlation for all items ranged from 0.3 to 0.5, and the internal consistency was good (Cronbach's α = 0.73). The area under the curve of EquiSed HHAG as a predictive diagnostic measure was 0.88. The sensitivity of the EquiSed calculated according to the cut-off point (score 7 of the sum of the EquiSed) determined by the receiver operating characteristic curve, was 96% and specificity was 83%. EquiSed has good intra- and interobserver reliabilities and is valid to evaluate tranquilization and sedation in horses.

## Introduction

The mortality related to general anesthesia in horses is high and represents 0.9% for elective surgeries (1). A current tendency exists to avoid general anesthesia when possible. Therefore, certain diagnostic and surgical procedures commonly use protocols based on constant rate infusions (CRIs) that provide consistent sedation, analgesia with minimal ataxia in the standing horse, thus ensuring the safety of the animal and those involved (2–4).

Scales with different scoring methods and stimuli have been proposed to determine the degree and quality of sedation of pharmacological protocols (4). Head height above the ground (HHAG) is an objective method expressed on the percentage change from baseline, considered as 100% (HHAG%). Reductions of the HHAG% by 50% or more represent a sufficiently sedated animal (2, 3, 5). Subjective scoring methods, such as visual analog (VAS) (6, 7), simple descriptive (SDS) (8, 9), numerical (NRS), and composite numerical scales (composite NRS) (10) are those that depend on the interpretation of the evaluator.

Studies from the early 1990s evaluated the quality of sedation using the composite NRS with mechanical, auditory, and visual stimuli (11–13). In order to produce mechanical stimuli, the use of pressure algometers (14) or tactile methods by touching an object inside the ears or on the coronary band (11–13, 15) has been reported. Auditory stimuli ranged from clapping behind (7, 11–13) or in front of the animal (15), sounds of scraping a spoon in a can (5), shaking a plastic gallon container filled with stones (14, 16) or the combination of auditory and sensory stimuli of a grooming machine connected and played in the lateral cervical region (17). Shaking a towel (7, 11–13) or opening an umbrella in front of the horse (5, 18) were forms of visual stimuli.

Postural instability and ataxia are subjective methods to evaluate sedation with the animal standing still (11), in the first case and by walking in a circular motion (5), or when getting over small obstacles (14, 16) in the second case. For ataxia, the VAS (2, 3), SDS (9, 19), and composite NRS have been described (7).

The use of reproducible and valid instruments is essential to guarantee the reliability and accuracy of measurements between different studies (20). Scale validation processes include content validity, training of observers, frequency distribution of scores, principal components analysis, intra- (repeatability) and interobserver (reproducibility) reliability, concurrent and construct validity (responsiveness), item-total correlation, internal consistency, receiver operating characteristic (ROC) curve, sensitivity, and specificity (20–26).

This study hypothesis that the proposed scale, based on stimuli and scales described in previous studies (2, 3, 7, 11–18, 27–31), accurately and reliably measures tranquilization and sedation in horses. Therefore, the current work aimed to develop and validate a composite scale of sedation in horses (EquiSed) applied to different pharmacological protocols and intensities of tranquilization and sedation.

## Materials and Methods

### Ethics Statement

The study was approved by the Ethics Committee on the Use of Animals for research at the School of Veterinary Medicine and Animal Science, São Paulo State University (UNESP), Botucatu, SP, Brazil, under protocol 2017/0051. This prospective, randomized, blinded, horizontal, and controlled study was carried out on the experimental farm of the same institution, to which the horses belonged. Their use was authorized by the person in charge of the area of equine culture (JNPPF). Phase I of the study was opportunistic with another study on equine standing sedation/antinociception (18) comparing different CRI protocols of detomidine alone or associated with methadone. Phase II was added to investigate the addition of two different doses of acepromazine under similar conditions of Phase I.

### Animals

In both Phases I and II, the same three male geldings and four female crossbred Quarter-horse and Appaloosa horses from the same herd were used (18), with a mean ± SD (range) age of 10 ± 0.95 (9–11) years and weight of 402 ± 21 (372–450) kg. Two weeks before the start of each phase of the study, all the horses were classified as healthy based on normal physical and laboratory examinations of blood count and biochemical analysis (urea, alkaline phosphatase, alanine aminotransferase, and gamma glutamyl transferase). The horses were kept on pasture and fed with hay and commercial feed once a day. The day before the study, the horse was separated and remained in covered facilities, with water *ad libitum* and access to the outside area. The solid fasting period was 2 h. Experimental protocols were performed for each horse on fixed days and periods (morning or afternoon) during the week to respect a drug washout period of 7 days. After the study, the horses were maintained at the farm of the institution and were used for riding and reproduction, the latter only if females.

The sample size was calculated by using the differences between the HHAG at baseline and maximal sedation, with a mean of 42.7% and SD of 11.8%. This was based, on a previously described pilot study (18), conducted with two horses undergoing detomidine and methadone sedation protocols (DL 2.5 μg/kg + 6.25 μg/kg/h; DH 5 μg/kg +12.5 μg/kg/h; DLM and DHM, 0.2 mg/kg + 0.05 mg/kg/h). The sample size was defined considering a type I error probability (α) of 0.05 and power (1–β) of 0.80. Based on this calculation, a sample size of four horses was required. The sample size was also corroborated by previous studies from the same group with the same animals (7) (https://www.statstodo.com/SSizUnpairedDiff_Pgm.php).

### Scale Development

The proposed scale was developed according to previously described stimuli that evaluate sedation in horses (Table 1) (11–13). For each item, specific response descriptions were assigned to each score from 0 to 3 (Table 1), where 0 corresponds to no sedation and 3 to maximum sedation. The sum of the items made up the scale, where 0 represents the absence of sedation and 18 the maximum sedation.

**Table 1:** EquiSed to evaluate the quality and degree of tranquilization and sedation in horses.

**Intensity of sedation**			
**Head Height Above Ground (HHAG%)**			**References**
Height from chin to floor. The height of the head for each horse is measured at baseline and considered as 100%. Height during sedation is calculated as a percentage compared to height at baseline.			(2, 4, 7, 12–15, 18)
**Stimuli performed**	**Response to stimuli**	**Scores**	**References**
*Touch the ear*			(11, 12)
Touch inside the ears with blunt-tipped material for 3 s	No response	3	
	Slight movement of the ear and/or head and/or neck	2	
	Intense movement of the ear and/or head and/or neck	1	
	Intense movement of the ear and head and/or neck and body movement	0	
*Press the coronary band of the thoracic limb*			(5, 11, 12)
Apply strong pressure for 3 s with blunt-tipped material on the coronary band of thoracic limb	No response	3	
	Moves the limb slowly without raising it	2	
	Raises the limb slowly	1	
	Raises the limb quickly before or when touched and/or moves the other limbs and/or head and/or trunk	0	
*Press the coronary band of pelvic limb*			(5, 11, 12)
Apply strong pressure for 3 s with blunt tipped material on the coronary band of pelvic limb	No response	3	
	Moves the limb slowly without raising it	2	
	Raises the limb slowly	1	
	Raises the limb quickly before or when touched and/or moves the other limbs and/or head and/or trunk	0	
*Postural instability*			(11–13, 15, 19, 31)
Observe the stationary animal and then forcefully push it laterally	Intense swaying, risk of falling down or falling down. Abducts (wide stance) the thoracic and/or pelvic limbs, and/or one limb misaligned or crossed	3	
	Moderate swaying. Thoracic and/or pelvic limbs abducted (wide stance), and/or one limb misaligned	2	
	No or slight swaying. One limb abducted (wide stance)	1	
	No swaying. Weight-bearing on all limbs, or resting one limb	0	
*Auditory*			(7, 11–13)
Response to loud hand clap behind the animal	No response	3	
	Slow movement of the head and/or neck and/or ear(s)	2	
	Rapid movement of the head and/or neck and/or ear(s)	1	
	Rapid movement of the head and/or neck and/or ear(s) and body movement	0	
*Visual*			(5)
Response to opening an umbrella in front of the animal	No response	3	
	Slight movement of the head and/or neck and/or ear(s)	2	
	Intense movement of the head and/or neck and/or ear(s)	1	
	Moves the head and/or neck and/or ear(s) and limb(s)	0	
Maximum possible sum of the EquiSed		18	

*EquiSed—Composite numerical scale of sedation in horses*.

### Data Collection

#### Phase I

The first Phase of the study was randomized (https://sorteador.com.br) with the main evaluator (MGM) unaware of the treatment received (18). The horses were weighed in a manual weighing scale and taken to the six-square meters experimental room for application of repellent, clipping the jugular areas, antisepsis of the region, and insertion and fixation of a 14-gauge catheter (G14 × 70 mm) in the left jugular vein to administer the drugs.

Before drug administration, the horses were placed to the containment stocks inside the experimental room, where they were fitted with the necessary equipment for the original study (18). Two video cameras installed inside the experimental stall were used to film the horse for viewing the animal as a whole, one in an oblique craniocaudal position, and another in an oblique caudocranial position.

After baseline data collection, one of the following intravenous (i.v.) treatments (bolus + CRI for 120 min) was administered: DL—detomidine low dose alone (2.5 μg/kg followed by a CRI at 6.25 μg/kg/h), DH—detomidine high dose alone (5 μg/kg followed by a CRI at 12.5 μg/kg/h), DLM—DL with methadone (2.5 μg/kg detomidine + 0.2 mg/kg methadone followed by CRIs of detomidine at 6.25 μg/kg/h and methadone at 0.05 mg/kg/h), and DHM—DH with methadone (5 μg/kg bwt detomidine + 0.2 mg/kg of methadone followed by CRIs of detomidine at 12.5 μg/kg/h and methadone at 0.05 mg/kg/h). Once the drug(s) *boli* were administered slowly by hand, CRIs were administered using two calibrated syringe drivers, one for each drug. The CRIs were administered for 2 h and the horses were kept in the stocks for 4 h due to the design of the pharmacokinetic phase (32) of the principal study (18).

All data were collected at baseline (before drug administration) and at 5, 15, 30, 60, 90, 120, 150, 180, 210, and 240 min after the start of the treatments. The HHAG was measured in cm with a scale fixed to the wall ~1.5 meters away from the horse. The HHAG for each horse from chin to floor was measured at baseline and considered as 100%. Height during tranquilization/sedation was calculated as a percentage compared to the height at baseline (2, 11). Afterwards, the filming and application of EquiSed stimuli started in the same order as the scale shown in Table 1. The EquiSed stimuli were applied *in situ* by the evaluator unaware of the treatments.

#### Phase II

This phase was neither covert nor the treatments were randomized at the *in situ* data collection, as it was included 11 months after Phase I. Horses were treated with i.v. acepromazine *boli* at low (ACPL 0.02 mg/kg) and high doses (ACPH 0.09 mg/kg). The same methodology regarding the filming, stimuli application and *in situ* data collection was used as in Phase I, except that the horses were kept in the stocks for only 120 min.

#### Video Selection and Evaluations

To validate EquiSed, four representative moments of the possible sedation intensities were defined: (i) baseline, (ii) peak sedation, (iii) intermediate sedation, and (iv) end of sedation (return to baseline).

Videos of Phase I at those representative moments were selected based on results obtained in a previous study: (18, 32). (i) the baseline time was before drug(s) were administered (T0), (ii) the peak of sedation was 120 min (T120) after the start of detomidine and immediately before the end of CRIs (iii) intermediate sedation was at 30 min after stopping the CRI(s), and (iv) the final moment of sedation was at 120 min after stopping the CRIs (T240—after starting the drug administration).

The selection of Phase II videos was based on the *in situ* results of the sedative effects of acepromazine. The moments (i) baseline, (ii) peak sedation at 60 min, (iii) intermediate sedation at 90 min, and (iv) end of sedation at 120 min after the administration of the acepromazine *boli* were selected.

In total, 168 videos (7 horses × 6 treatments × 4 representative moments) were selected to be evaluated, each lasting ~45 s.

Three Diplomates from the European College of Veterinary Anesthesia and Analgesia (ECVAA) from different institutions (MGM; SKR and SS), together with the responsible researcher (ARO) evaluated the videos, independently. Evaluator 1 (E1—ARO) had 7 years of experience in veterinary anaesthesiology and sedation in horses, while evaluators 2, 3, and 4 (E2—MGM, E3—SKR, and E4—SS) had an experience of 15, 17, and 18 years, respectively. Evaluator 2 was certified by ECVAA, in 2014 and E3 and E4 in 2009. The evaluators became familiar with the EquiSed items through tutorial videos demonstrating each score Supplementary Table 1. For training, the evaluators assessed 10% of the total videos (16 videos, four from each sedation moment) randomly, in two different periods, with an interval of 1 month. Spearman's correlation, intra- and interobserver reliability values in this training phase were ≥ 80%, indicating that the evaluators were able to identify the intensity of sedation similarly.

Once the training phase was completed, the main evaluations took place after 2 months. The 168 videos were randomized and made available on a Google Drive digital platform (UNESP license—G Suit for Education) in two independent folders (www.random.org), so that the evaluators could allocate, unaware of the different sedation moments, the scores corresponding to each video in two phases with intervals of 1 month. Before beginning the evaluations, the evaluators read the instructions for the analysis and completion of the evaluation worksheets. Instructions included: (1) how to evaluate the scale and its descriptors to guarantee that the stimuli responses were clear, and (2) how to complete the worksheets following the evaluation of the videos. The evaluators were instructed to watch the videos as many times as they deemed necessary to complete the spreadsheets and to evaluate the videos for up to 1 h a day so that fatigue would not interfere with the evaluation. The spreadsheet was filled out in the order NRS, SDS, VAS for general sedation, VAS for postural instability, and EquiSed, in the same order in which the stimuli were applied. The HHAG values used in this manuscript were only collected *in situ* by the main evaluator (18) and transformed into HHAG% for data analysis for convenience.

### Statistical Analysis

#### Content Validity

The content validation of the EquiSed was performed in three stages. First, the inclusion of stimuli described in previous studies evaluating sedation in horses and their responses (Table 1). Second, the critical analysis by the evaluators ARO, MGM, SKR, and SS, regarding the interpretation of the EquiSed. Finally, the analysis of the relevance, in which attribution of the importance of each item was made as previously described in processes for validating pain scales in other species (23, 25, 26). For this last stage, three external evaluators (MOT, FAO, and CL), all veterinary anaesthesiologists with more than 10 years of experience in the field assigned the relevance of each scale item from +1, for relevant, 0, for not being able to give an opinion, and −1, for irrelevant. The mean relevance of each item was calculated, and those with a value ≥ 0.5 remained in the EquiSed scale (Table 1).

The relevance of all the items of EquiSed identified in content validity indicated that the main videos scoring could be performed by the four evaluators, with these scoring data. Therefore, all the following analysis were performed, except for the HHAG%, that was not collected by video analysis.

All the following analyses were performed with data from all evaluators, treatments, first and second evaluations, and grouped moments, except where reported below.

#### Reliability

To evaluate intraobserver (repeatability; comparison of data from the first and second evaluations of the videos) and interobserver reliability (reproducibility; comparison of data from the first and second evaluations of the videos between all the evaluators, also called interobserver agreement matrix), we used the weighted kappa coefficient (K_w_) with a 95% confidence interval (CI) for the NRS, SDS, and each item of the EquiSed. The disagreements were weighted according to their distance to the square of perfect agreement. The intraclass correlation coefficient (ICC) of the agreement type with 95% CI was used to analyse the sum of the EquiSed and the ICC of the consistency type with 95% CI to analyse the VAS sedation and VAS postural instability. Reliability for the weighted Kappa and ICC (CI) was considered to be very good if 0.81–1.0; good if 0.61–0.80; moderate if 0.41–0.60; reasonable if 0.21–0.40; and poor if <0.20 (33, 34).

#### Concurrent Criteria Validation

The concurrent criterion validity was based on Spearman's correlation between the sum of the EquiSed *vs*. the NRS, SDS, and VAS scales (20, 26) and the HHAG%. To measure the correlation between the EquiSed and HHAG%, treatments were grouped into tranquilization (ACPL + ACPH), low dose detomidine (DL + DLM), and high dose detomidine (DH + DHM), to differentiate the correlation between acepromazine and the other detomidine treatments.

#### Construct Validity (Responsiveness)

No data presented normal distribution according to Shapiro-Wilk test. Therefore, the Friedman test was used for responsiveness comparisons over time (baseline, peak sedation, intermediate sedation, and end of sedation), and the Kruskal-Wallis test for comparisons between treatments at each time, with the Dunn's post-test in both cases. For all variables, data from all grouped evaluators were used, except for the HHAG%, which used data from the study previously published with the evaluations *in situ* (18).

#### Principal Component Analysis (PCA)

The PCA is a multiple association test that evaluates the association of all items of the scale with each other (35). This analysis was performed based on the correlation matrix. The Kaiser criterion (36) was used to select dimensions with eigenvalues > 1, variance > 20, and the items within dimensions with load factor ≥ 0.50 or ≤ −0.50.

#### Item-Total Correlation

The item-total correlation was performed to identify the scale homogeneity, through Spearman's correlation between the score attributed to each item *vs*. the sum of the EquiSed excluding the item analyzed. Values were accepted between 0.3 and 0.7 (33).

#### Internal Consistency

The internal consistency of the scale was calculated using Cronbach's α coefficient (37), which determines how much the items on the scale correlate with each other. Minimum acceptable values were considered between 0.60 and 0.64, acceptable 0.65–0.69, good 0.70–0.74, very good 0.75–0.80, and excellent above 0.80 (38).

#### Sensitivity and Specificity

To determine sensitivity and specificity, data were transformed into dichotomous values for the presence (1, corresponding to score 1, 2, or 3) or absence (0, corresponding to score 0) of representative sedation scores at peak sedation times and at baseline times, respectively. Specificity is generated by the relationship between the total true negative results (horses not sedated—score 0) in relation to the total number of evaluations at baseline when the scores were not expected to indicate sedation. Sensitivity was calculated by the ratio of true positives (horses with scores 1, 2, or 3) in relation to the total number of assessments at peak sedation. Both were interpreted as excellent, when 95–100%, good, when 85–94.9%, moderate when 70–84.9%, and non-sensitive or specific, when <70% (24, 39). The sensitivity and specificity of the total score of the scale was performed based on the cut-off of the area under the receiver operating characteristic (ROC) curve described below. Horses showing scores > 7 at the peak of sedation were considered sufficiently sedated and horses showing scores ≤ 7 at baseline were considered non-sedated.

#### ROC Curve and Cut-Off Point for Sedation

Scores of horses treated with DH and DHM were used for these calculations. The peak of sedation and baseline time points were used to calculate sensitivity (true positives or truly sedated horses) and specificity (true negatives or no sedated horses), respectively. The HHAG% ≤ 50% was used as a predictive value to consider truly sedated horses to build the ROC curve. The ROC curve is the graphical representation of the relationship between true positives (sensitivity) and false positives (1-specificity). Cut-off point was defined by the Youden index (YI) and its diagnostic uncertainty zone. The YI is the greatest coincident point of sensitivity and specificity, determined by the ROC curve. The gray zone (diagnostic uncertainty zone) indicates diagnostic accuracy and is provided by calculating 95% CI by replicating the original ROC curve 1,001 times by the bootstrap method and by the interval between sensitivity and specificity. Gray zone was considered the greatest interval of these two methods (33).

#### Frequency Distribution of Scores

The frequency of distribution of scores 0, 1, 2, and 3 of each item at each time in the grouped treatments (ACPL + ACPH, DL + DLM. and DH + DHM) was analyzed using descriptive statistics.

Statistical analysis was performed using R software in the RStudio integrated development environment (RStudio Team-−2016) and Microsoft Office® (Excel−2019).

## Results

The EquiSed showed intra- and interobserver reliability, content, criterion, and construct validity and adequate item-total correlation and internal consistency.

### Training

In the training stage, the evaluators presented excellent interobserver reliability (82–93%) and intraobserver reliability (88, 82, 91, and 85% for E1–E4, respectively).

### Content Validity

The characteristics described based on previous studies, and the semantic clarity of the items' descriptions were approved by the evaluators of the study (Table 1).

All items on the scale had a sum >0.5 for the mean degree of relevance for the three external veterinary anaesthesiologists. Thus, there were no changes in the content of the scale.

### Intraobserver Reliability (Repeatability)

Results of intraobserver reliability are presented in Table 2. The repeatability of the EquiSed varied from good to very good for thoracic limbs and visual items (CI 0.69–0.95). The other items were reasonable to very good (CI 0.37–0.93). The repeatability of the sum of the EquiSed was very good (CI 0.84–0.94), the NRS varied from moderate to very good (CI 0.67–0.93), the VAS sedation from moderate to very good (CI 0.62–0.92), the VAS postural instability from moderate to very good (CI 0.54–0.87), and the SDS from moderate to very good (CI 0.47–0.83).

**Table 2:** Intraobserver reliability of the NRS, SDS, EquiSed items, sum of EquiSed, VAS sedation, and VAS postural instability scores in horses treated with acepromazine or detomidine alone or associated with methadone.

**EquiSed items**	**E1**		**E2**		**E3**		**E4**	
	***k_***w***_***	**CI**	***k_***w***_***	**CI**	***k_***w***_***	**CI**	***k_***w***_***	**CI**
NRS	**0.80**	0.73–0.85	**0.89**	0.85–0.93	**0.90**	0.85–0.93	**0.75**	0.67–0.82
SDS	**0.73**	0.64–0.82	**0.69**	0.57–0.78	**0.75**	0.65–0.83	**0.55**	0.47–0.64
Ears	**0.67**	0.57–0.76	**0.78**	0.68–0.86	**0.77**	0.70–0.82	**0.73**	0.65–0.81
Thoracic limb	**0.91**	0.82–0.95	**0.89**	0.75–0.95	**0.90**	0.81–0.95	**0.92**	0.87–0.95
Pelvic limb	**0.81**	0.59–0.91	**0.83**	0.70–0.92	**0.86**	0.75–0.93	**0.80**	0.71–0.89
Postural instability	**0.69**	0.57–0.78	**0.58**	0.46–0.68	**0.78**	0.71–0.84	**0.53**	0.39–0.64
Auditory	**0.62**	0.51–0.71	**0.62**	0.46–0.73	**0.59**	0.47–0.68	**0.58**	0.37–0.70
Visual	**0.82**	0.76–0.87	**0.85**	0.78–0.90	**0.84**	0.77–0.90	**0.76**	0.69–0.87
**ICC (agreement)**								
	**ICC**	CI	**ICC**	CI	**ICC**	CI	**ICC**	CI
Sum of the EquiSed	**0.89**	0.84–0.92	**0.91**	0.87–0.94	**0.91**	0.88–0.94	**0.89**	0.84–0.92
**ICC (consistency)**								
	**ICC**	CI	**ICC**	CI	**ICC**	CI	**ICC**	CI
VAS sedation	**0.76**	0.69–0.82	**0.89**	0.85–0.92	**0.89**	0.86–0.92	**0.71**	0.62–0.78
VAS postural instability	**0.70**	0.62–0.77	**0.64**	0.54–0.72	**0.83**	0.78–0.87	**0.56**	0.45–0.66

*EquiSed—Numerical composite sedation scale in horses, NRS—Numerical rating scale, SDS—Simple descriptive scale, VAS—Visual analog scale. E1, Evaluator 1; E2, Evaluator 2; E3, Evaluator 3; E4, Evaluator 4. Interpretation of weighted Kappa (k_w_) and ICC (intraclass correlation coefficient)—very good if 0.81–1.0; good if 0.61–0.80; moderate if 0.41–0.60; reasonable if 0.21–0.40; and poor if <0.20. CI (confidence interval) (33, 34). Bold values represent the Kw and ICC of intraobserver reliability*.

### Interobserver Agreement Matrix (Reproducibility)

Results of interobserver reliability are presented in Table 3. The individual items showed poor to very good interobserver reliability (CI 0.18–0.91). The sum of EquiSed showed moderate to very good interobserver reliability (CI 0.45–0.88). The reliability of the NRS and VAS sedation ranged from moderate to very good (CI 0.50–0.85). The SDS and VAS of postural instability showed reasonable to good reliability (CI 0.20–0.66), respectively.

**Table 3:** Interobserver agreement matrix of the sedation scores of the NRS, SDS, EquiSed items, sum of EquiSed, VAS sedation, and VAS postural instability in horses treated with acepromazine or detomidine alone or associated with methadone.

**Evaluator**	**E1**		**E2**		**E3**	
	***k_***w***_***	**CI**	***k_***w***_***	**CI**	***k_***w***_***	**CI**
**NRS**						
**E2**	**0.72**	0.66–0.77				
**E3**	**0.75**	0.70–0.80	**0.78**	0.73–0.82		
**E4**	**0.67**	0.60–0.72	**0.79**	0.74–0.84	**0.81**	0.76–0.85
**SDS**						
**E2**	**0.41**	0.34–0.47				
**E3**	**0.34**	0.28–0.40	**0.43**	0.35–0.50		
**E4**	**0.43**	0.36–0.50	**0.60**	0.52–0.66	**0.59**	0.51–0.64
**Touch the ears**						
**E2**	**0.66**	0.59–0.73				
**E3**	**0.63**	0.56–0.70	**0.61**	0.54–0.67		
**E4**	**0.54**	0.47–0.61	**0.58**	0.51–0.65	**0.69**	0.62–0.74
**Pressure on the thoracic limb**						
**E2**	**0.92**	0.87–0.94				
**E3**	**0.92**	0.97–0.94	**0.92**	0.86–0.95		
**E4**	**0.90**	0.86–0.93	**0.92**	0.87–0.95	**0.91**	0.86–0.94
**Pressure on the pelvic limb**						
**E2**	**0.77**	0.65–0.85				
**E3**	**0.82**	0.73–0.89	**0.85**	0.78–0.91		
**E4**	**0.68**	0.56–0.76	**0.75**	0.66–0.83	**0.78**	0.71–0.85
**Postural instability**						
**E2**	**0.47**	0.39–0.54				
**E3**	**0.54**	0.47–0.61	**0.50**	0.40–0.57		
**E4**	**0.40**	0.31–0.49	**0.43**	0.33–0.51	**0.43**	0.34–0.51
**Auditory**						
**E2**	**0.47**	0.40–0.54				
**E3**	**0.52**	0.44–0.59	**0.58**	0.50–0.66		
**E4**	**0.31**	0.26–0.38	**0.26**	0.18–0.33	**0.37**	0.29–0.44
**Visual**						
**E2**	**0.74**	0.68–0.78				
**E3**	**0.64**	0.57–0.70	**0.72**	0.66–0.77		
**E4**	**0.38**	0.33–0.44	**0.46**	0.41–0.51	**0.55**	0.49–0.61
	**ICC**	CI	**ICC**	CI	**ICC**	CI
**EquiSed**						
**E2**	**0.81**	0.77–0.85				
**E3**	**0.78**	0.74–0.82	**0.86**	0.83–0.88		
**E4**	**0.53**	0.45–0.61	**0.72**	0.66–0.77	**0.78**	0.73–0.82
**VAS sedation**						
**E2**	**0.65**	0.58–0.71				
**E3**	**0.75**	0.70–0.80	**0.74**	0.69–0.79		
**E4**	**0.58**	0.50–0.64	**0.78**	0.74–0.82	**0.75**	0.70–0.79
**VAS postural instability**						
**E2**	**0.44**	0.35–0.52				
**E3**	**0.59**	0.52–0.66	**0.30**	0.20–0.39		
**E4**	**0.42**	0.33–0.50	**0.44**	0.35–0.52	**0.32**	0.22–0.41

*EquiSed—Numerical composite sedation scale in horses, NRS—Numerical rating scale, SDS—Simple descriptive scale, VAS—Visual analog scale. E1, Evaluator 1; E2, Evaluator 2; E3, Evaluator 3; E4, Evaluator 4. Interpretation of weighted Kappa (k_w_) and ICC—very good if 0.81–1.0; good if 0.61–0.80; moderate if 0.41–0.60; reasonable if 0.21–0.40; and poor if <0.20. CI (confidence interval) (33, 34). Bold values represent the Kw and ICC of intraobserver reliability*.

### Concurrent Criterion Validity

The correlations between the sum of the EquiSed and all other scales were high when all the treatments were analyzed combined, except for the HHAG%, which presented a medium correlation, just as in low (DL + DLM) and high (DH + DHM) detomidine, and a low correlation for tranquilization (ACPL + ACPH) (Table 4).

**Table 4:** Correlation between the EquiSed with the NRS, SDS, and VAS sedation registered by analyzing the videos and with the HHAG% recorded *in situ* in horses treated with acepromazine or detomidine alone or associated with methadone.

**Scales/EquiSed**	**Treatments**	**Grouped moments**
NRS	Tranquilization (ACPL + ACPH)	0.65 CI (0.63–0.73)
	Low detomidine (DL+ DLM)	0.69 CI (0.68–0.76)
	High detomidine (DH + DHM)	**0.84** CI (0.82–0.88)
	All treatments	**0.77** CI (0.74–0.79)
SDS	Tranquilization (ACPL + ACPH)	0.57 CI (0.51–0.64)
	Low detomidine (DL+ DLM)	0.67 CI (0.63–0.74)
	High detomidine (DH + DHM)	**0.78** CI (0.77–0.83)
	All treatments	**0.70** CI (0.67–0.73)
VAS sedation	Tranquilization (ACPL + ACPH)	0.64 CI (0.61–0.71)
	Low detomidine (DL+ DLM)	0.68 CI (0.66–0.77)
	High detomidine (DH + DHM)	**0.84** CI (0.83–0.88)
	All treatments	**0.76** CI (0.73–0.78)
HHAG%	Tranquilization (ACPL + ACPH)	−0.14 CI (−0.07–0.43)
	Low detomidine (DL+ DLM)	−0.40 CI (−0.28 to −0.68)
	High detomidine (DH + DHM)	−0.69 CI (−0.66 to −0.85)
	All treatments	−0.52 CI (−0.65 to −0.44)

*EquiSed—Numerical composite sedation scale in horses, NRS—Numerical rating scale, SDS—Simple descriptive scale, VAS sedation—Visual analog scale of sedation. HHAG%, Head height above the ground %. Interpretation of Spearman's correlation−0–0.35–low correlation; 0.35–0.7—medium correlation; 0.7–1.0—high correlation. Bold values correlation> 70 (20, 33, 40)*.

### Construct Validity (Responsiveness)

Results of responsiveness are presented in Table 5. The postural instability and auditory stimuli were responsive, as shown by the difference between the baseline and the peak of sedation value, with all treatments. The touch in the ears and the visual stimuli showed responsiveness for all detomidine treatments, but not for the two doses of acepromazine. The pressure stimuli on the thoracic and pelvic limbs were responsive for the high doses of detomidine (DH + DHM) only. When all treatments were grouped, all items showed responsiveness.

**Table 5:** Responsiveness during and between treatments of the EquiSed, NRS, SDS, VAS sedation, and HHAG% in horses treated with acepromazine or detomidine alone or associated with methadone.

		**Moments**							
**Scales**		**Baseline**		**Peak of sedation**		**Intermediate sedation**		**End of sedation**	
	**Items**	**Median**	**Amplitude**	**Median**	**Amplitude**	**Median**	**Amplitude**	**Median**	**Amplitude**
NRS	ACPL	2^cAB^	1–5	2^bcC^	1–6	3^aB^	1–6	2^bB^	1–5
	ACPH	1^bB^	0–6	4^aB^	1–7	4^aB^	1–7	4^aA^	1–7
	DL	2^bcA^	1–7	5^aB^	2–9	3^bB^	1–8	2^cBC^	1–5
	DLM	1^cB^	0–6	5^aB^	1–8	3^bB^	1–7	2^cC^	1–4
	DH	2^cAB^	0–6	7^aA^	5–9	4^bA^	2–8	2^cC^	1–5
	DHM	2^cAB^	1–5	8^aA^	4–10	6^bA^	2–8	2^cBC^	1–5
	All	2^d^	0–7	5^a^	1–10	4^b^	1–8	2^c^	1–7
SDS	ACPL	1^b^	0–2	1^abC^	0–2	1^aC^	0–2	1^abAB^	0–2
	ACPH	0^b^	0–2	1^aB^	0–2	1^aBC^	0–2	1^aA^	0–2
	DL	1^bc^	0–2	2^aB^	0–3	1^bC^	0–2	1^cB^	0–2
	DLM	0^c^	0–2	2^aB^	0–3	1^bC^	0–2	1^cB^	0–1
	DH	1^c^	0–2	2^aA^	1–3	1^bAB^	0–2	1^cB^	0–2
	DHM	1^b^	0–2	2^aA^	1–3	2^aA^	0–3	1^bB^	0–2
	All	1^c^	0–2	2^a^	0–3	1^b^	0–3	1^c^	0–2
Ears	ACPL	1	0–2	1^C^	0–2	1^B^	0–3	1	0–2
	ACPH	1	0–3	1^B^	0–3	1^B^	0–3	1	0–3
	DL	1^b^	0–2	1^aB^	1–3	1^abB^	0–2	1^b^	0–3
	DLM	1^b^	0–3	1^aB^	0–3	1^abB^	0–3	1^b^	0–3
	DH	1^b^	0–3	2^aA^	0–3	1^bAB^	0–2	1^b^	0–3
	DHM	1^b^	0–2	2^aA^	1–3	2^aA^	0–3	1^b^	0–2
	All	1^c^	0–3	2^a^	0–3	1^b^	0–3	1^c^	0–3
Thoracic	ACPL	0	0–3	0^C^	0–3	0^AB^	0–3	0^A^	0–3
limb	ACPH	0	0–3	0^ABC^	0–3	0^A^	0–3	0^A^	0–3
	DL	0	0–3	0^C^	0–3	0^AB^	0–3	0^AB^	0–2
	DLM	0	0–2	0^BC^	0–3	0^AB^	0–1	0^AB^	0–2
	DH	0^b^	0–2	3^aAB^	0–3	0^bB^	0–1	0^bB^	0–1
	DHM	0^b^	0–1	2^aA^	0–3	0^bAB^	0–2	0^bB^	0–1
	All	0^b^	0–3	0^a^	0–3	0^b^	0–3	0^b^	0–3
Pelvic	ACPL	0	0–1	0^C^	0–2	0^AB^	0–2	0^AB^	0–2
limb	ACPH	0	0–2	0^C^	0–1	0^B^	0–2	0^AB^	0–2
	DL	0	0–1	0^C^	0–1	0^B^	0–1	0^B^	0–0
	DLM	0^b^	0–1	0^aBC^	0–1	0^abB^	0–1	0^bB^	0–1
	DH	0^b^	0–1	1^aAB^	0–2	0^bAB^	0–1	0^bB^	0–1
	DHM	0^b^	0–1	2^aA^	0–3	0^bA^	0–2	0^bA^	0–1
	All	0^b^	0–2	0^a^	0–3	0^b^	0–2	0^b^	0–2
Postural	ACPL	0^bC^	0–1	1^aC^	0–2	1^aABC^	0–2	1^aAB^	0–2
instability	ACPH	0^bBC^	0–1	1^aBC^	0–2	1^aBC^	0–2	1^aA^	0–2
	DL	1^abA^	0–1	1^aB^	0–2	1^abBC^	0–1	0^bBC^	0–2
	DLM	0^bABC^	0–2	1^aB^	0–2	1^bB^	0–1	0^bC^	0–1
	DH	1^cAB^	0–1	2^aA^	0–2	1^bAC^	0–2	0^cBC^	0–1
	DHM	0^cABC^	0–2	2^aA^	0–3	1^bA^	0–2	0^cBC^	0–2
	All	0^c^	0–2	1^a^	0–3	1^b^	0–2	0^c^	0–2
Auditory	ACPL	2^b^	0–3	2^aC^	1–3	2^aB^	1–3	2^abAB^	1–3
	ACPH	2^b^	1–3	2^aBC^	1–3	2^aB^	0–3	2^aAB^	1–3
	DL	2^b^	0–3	3^aA^	2–3	2^abB^	1–3	2^bB^	1–3
	DLM	2^b^	0–3	3^aA^	1–3	2^aB^	1–3	2^bAB^	1–3
	DH	2^b^	1–3	3^aA^	1–3	3^aA^	1–3	2^bAB^	0–3
	DHM	2^b^	0–3	3^aAB^	1–3	3^aA^	2–3	2^bA^	1–3
	All	2^c^	0–3	3^a^	1–3	2^a^	0–3	2^b^	0–3
Visual	ACPL	1	0–3	1^C^	1–3	1^C^	1–3	2^AB^	0–3
	ACPH	1^b^	0–3	2^aBC^	1–3	2^aBC^	0–3	2^aA^	0–3
	DL	1^c^	0–2	2^aB^	0–3	2^abBC^	0–3	1^bcB^	0–3
	DLM	1^b^	0–3	2^aB^	0–3	2^aAB^	0–3	1^bB^	0–3
	DH	2^b^	1–3	3^aA^	1–3	3^aAB^	1–3	2^bAB^	0–3
	DHM	2^b^	0–3	3^aA^	2–3	3^aA^	1–3	1^bB^	0–2
	All	1^c^	0–3	2^a^	0–3	2^a^	0–3	2^b^	0–3
EquiSed	ACPL	5^b^	1–10	6^aC^	3–12	6^aB^	3–14	6^aAB^	2–12
	ACPH	4,5^b^	1–15	7^aB^	3–15	7^aB^	2–13	7^aA^	3–14
	DL	5,5^c^	2–12	7^aB^	5–14	6^bB^	2–12	5^cBC^	2–9
	DLM	4^c^	1–10	7^aB^	3–14	7^bB^	1–11	4,5^cC^	1–8
	DH	6^c^	2–10	11^aA^	7–16	8^bAB^	3–11	5^cBC^	2–9
	DHM	5^c^	2–11	12^aA^	9–16	8,5^bA^	5–15	5^cBC^	2–10
	All	5^d^	1–15	9^a^	3–16	7^b^	1–15	5^c^	1–14
VAS sedation	ACPL	7^c^	0–50	14b^cC^	0–59	24^aB^	0–62	19b^AB^	0–51
	ACPH	6^b^	0–60	36^aB^	0–64	31^aB^	0–67	28^aA^	0–77
	DL	15^bc^	0–61	47^aB^	2–83	22^bB^	0–73	10^cBC^	0–56
	DLM	6^c^	0–52	48^aB^	0–82	22^bB^	0–86	9^cC^	0–36
	DH	6^c^	0–52	70^aA^	44–99	40^bA^	14–81	7^cC^	0–46
	DHM	7^c^	0–47	76^aA^	31–99	55^bA^	4–80	11^cBC^	0–54
	All	7^d^	0–61	49^a^	0–99	32^b^	0–86	14^c^	0–77
VAS postural instability	ACPL	0^cB^	0–16	3^bcD^	0–31	10^aBC^	0–53	5a^bAB^	0–30
	ACPH	0^bB^	0–22	12^aC^	0–65	10^aBC^	0–47	10^aA^	0–66
	DL	7^bA^	0–30	12^aC^	0–74	7^bC^	0–41	2^bB^	0–43
	DLM	1^bcAB^	0–30	17^aBC^	0–62	6^bC^	0–39	0^cB^	0–25
	DH	0^cAB^	0–24	28^aA^	0–85	15^bAB^	0–77	3^cB^	0–22
	DHM	1^cAB^	0–37	41^aAB^	7–92	15^bA^	0–69	2^cAB^	0–51
	All	0.5^c^	0–37	15^a^	0–92	10^b^	0–77	3^c^	0–66
Height Head Above the Ground%^*^	ACPL	100^a^	100–100	89^abA^	27–100	84^bAB^	81–90	95^ab^	71–103
	ACPH	100^a^	100–100	66^bAB^	53–91	82^abAB^	50–102	92^ab^	68–105
	DL	100^a^	100–100	53^bAB^	22–71	93^abA^	44–105	103^a^	83–110
	DLM	100^a^	100–100	64^bAB^	29–89	85^abAB^	47–95	100^a^	96–107
	DH	100^a^	100–100	27^bB^	18–47	60^abB^	27–80	100^a^	86–105
	DHM	100^a^	100–100	29^bB^	14–74	68^abAB^	26–95	102^a^	84–147
	All	100^a^	100–100	53^b^	14–100	82^b^	26–105	100^a^	68–147

EquiSed—Numerical composite sedation scale in horses, NRS—Numerical rating scale, SDS—Simple descriptive scale, VAS—Visual analog scale; ACPL + ACPH (acepromazine in low and high doses); DL + DLM (detomidine in low and high doses); DH + DHM (detomidine in low and high doses);

* *data obtained from a previously published article (18). Different lower-case letters represent statistical differences over time (p < 0.05) (a> b> c). Different capital letters represent statistical differences between treatments (p < 0.05) (A>B>C)*.

**Figure 1:**
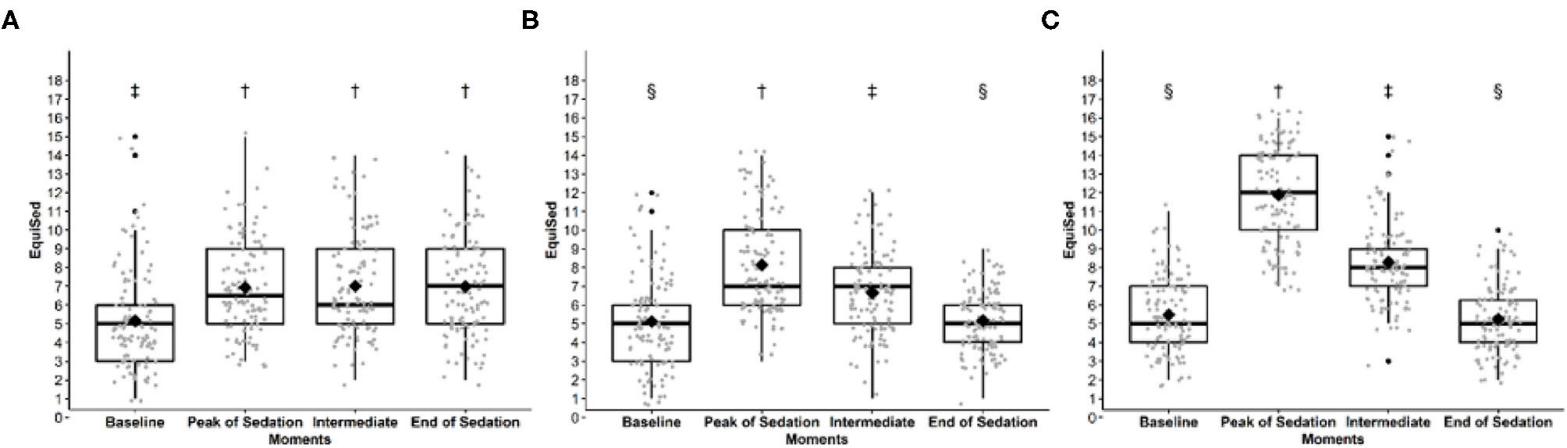
EquiSed scores before and after **(A)** tranquilization (ACPL + ACPH), **(B)** low detomidine (DL + DLM) and **(C)** high detomidine (DH + DHM). ACPL + ACPH—acepromazine in low and high doses; DL + DLM—detomidine in low dose and associated with methadone; DH + DHM—detomidine in high dose and associated with methadone. Baseline—baseline moment; Peak of sedation—peak of sedation; Intermediate—intermediate sedation; and end of sedation. Different symbols indicate significant differences between scores (*p* < 0.05). † > ‡> §.

All scales were responsive in all treatments, demonstrating higher values at peak sedation or intermediate sedation compared to baseline, except for the HHAG% which showed lower values at peak sedation or intermediate sedation compared to baseline (Table 5, Figure 1). When all treatments were grouped, scores on all scales were higher at the peak sedation and intermediate sedation in relation to the baseline and final moment of sedation. All the scales were able to differentiate the peak sedation from intermediate sedation, except for the HHAG%.

For horses receiving acepromazine, the scores of all scales except for HHAG% were higher at the final moment compared to baseline (Table 5, Figure 1).

Differences between treatments at peak of sedation were detected by all scales, except HHAG%. Sedation scores were highest with DH, followed by DL and ACP. The addition of methadone did not increase sedation.

### Principal Component Analysis (PCA)

The analysis of multiple associations by principal components defined the items with load factor of ≥ 50% (Table 6), acceptable according to the Kaiser criteria (36).

**Table 6:** Load values, eigenvalues, and variance of EquiSed items by the principal component analysis.

**EquiSed Items**	**Load factors in Dimension 1**	**Load factors in Dimension 2**
Touch the ears	**0.67**	0.07
Pressure on the thoracic limb	**0.71**	0.50
Pressure on the pelvic limb	**0.72**	0.36
Postural Instability	**0.68**	−0.17
Auditory stimulus	0.48	–**0.76**
Visual stimulus	**0.68**	−0.27
**Eigenvalue**	**2.62**	**1.07**
**Variance**	**43.64**	17.84

*EquiSed—Numerical composite sedation scale in horses. Eigenvalues> 1, variance > 20 and load factor ≥ 0.50 or ≤ −0.50 are approved (36). Load factors represent the correlation of items with the scale and how much each item contributes to each factor. Bold values represent load factor ≥ 0.50 or ≤ −0.50*.

**Figure 2:**
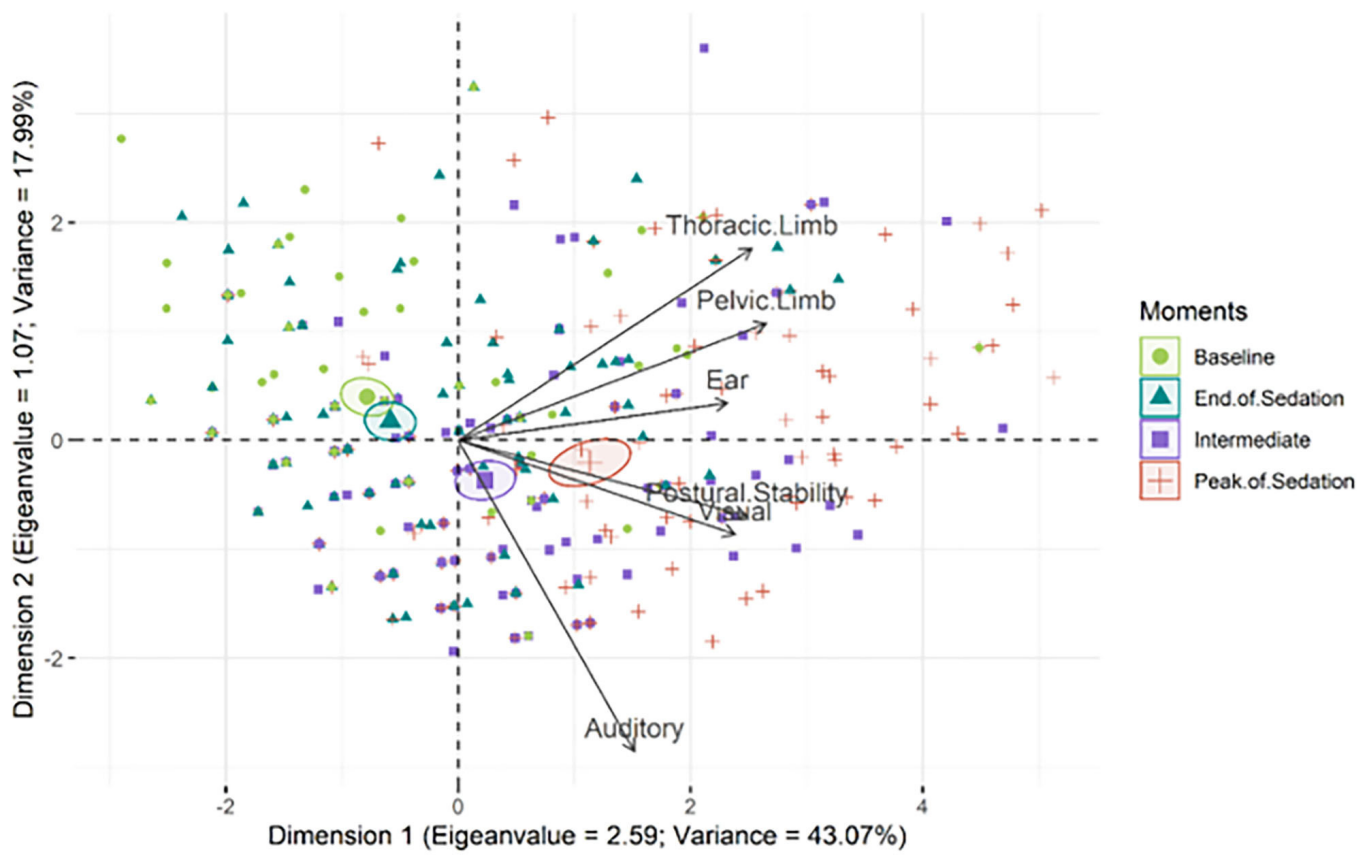
Biplot of the principal component analysis of the EquiSed. Confidence ellipses were built according to the moments before and after sedation. Baseline (green); peak of sedation (red); intermediate sedation (purple); end of sedation (blue). The ellipses on the left represent the absence or end of sedation and on the right represent the peak of sedation or intermediate sedation. The moments of greatest sedation (intermediate and peak) influence all the items on the scale since their vectors are directed to these ellipses. Each isolated symbol represents each assessment of each horse at each moment.

**Figure 3:**
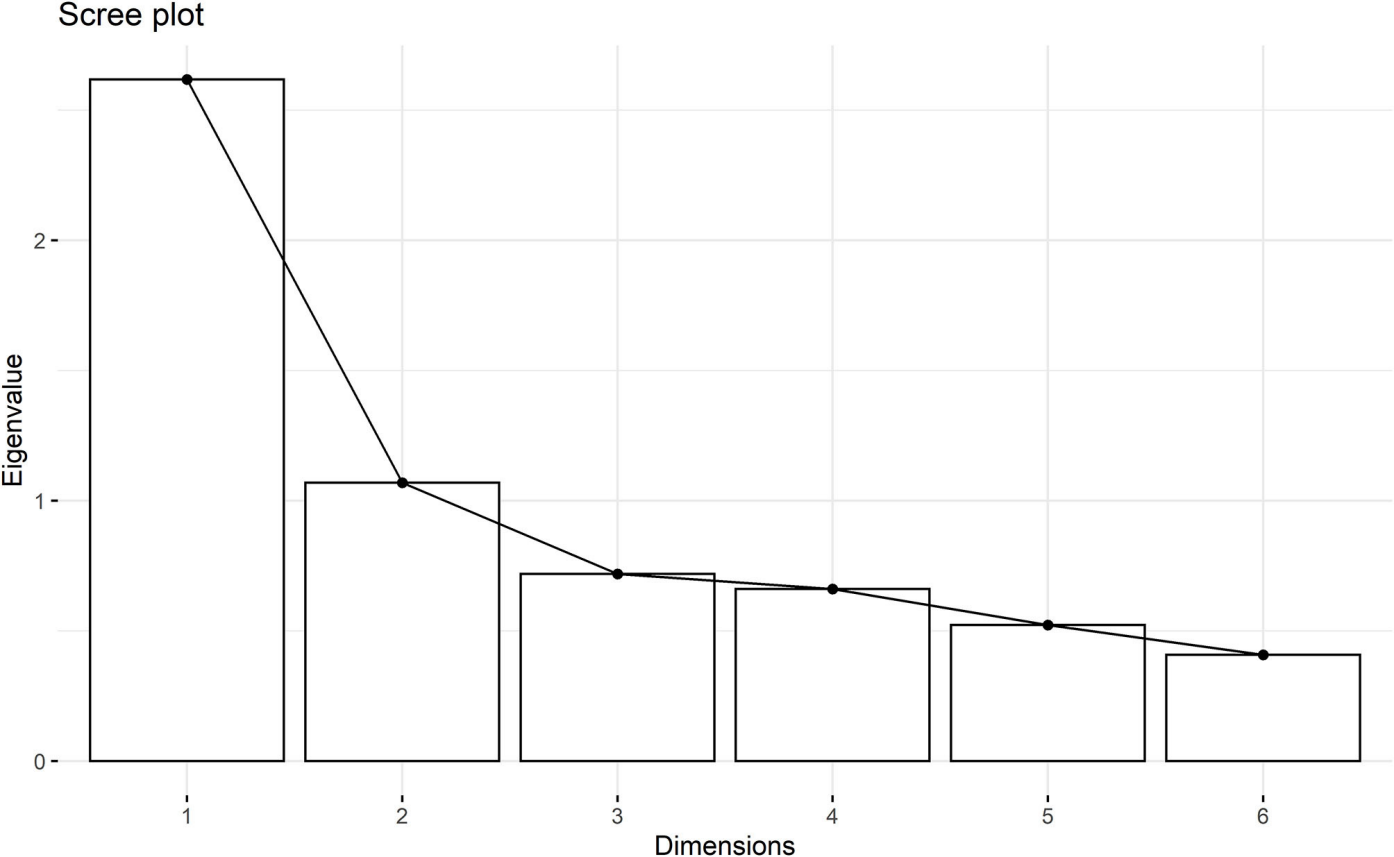
Histogram of eigenvalues of each dimension of the principal component analysis. First dimensions represent the majority of the items of Equised with eigenvalues > 1.0.

All items on the scale have the same vectorial direction to identify sedated animals (Figure 2). The histogram of each eigenvalue is represented on Figure 3.

### Item-Total Correlation and Internal Consistency

The item-total correlation ranged from 0.31 to 0.54, so all items (0.3–0.7) were accepted (Table 7) (33). The internal consistency was good for the total score of the scale and acceptable or good when excluding each item.

**Table 7:** Spearman's item-total correlation, internal consistency, sensitivity, and specificity of EquiSed.

**EquiSed items**	**Item-total correlation**	**Internal consistency**	**Sensitivity (CI)**	**Specificity (CI)**
**All items**	**Excluding each item below**	**0.73**	**0.96 (0.93–0.99)**	**0.83 (0.76–0.89)**
Touch the ears	**0.50**	**0.68**	**93 (91–96)**	16 (12–20)
Pressure on the thoracic limb	**0.49**	**0.69**	42 (38–48)	**74 (69–80)**
Pressure on the pelvic limb	**0.54**	**0.68**	39 (34–44)	**91 (88–94)**
Postural instability	**0.50**	**0.68**	**78 (74–82)**	62 (57–67)
Auditory stimulus	**0.31**	**0.73**	**100 (100–100)**	1 (1–1)
Visual stimulus	**0.49**	**0.68**	**100 (99–100)**	8 (6–12)

*EquiSed—Numerical composite sedation scale in horses. Interpretation of the Spearman item-total correlation (r_s_)—values between 0.3 and 0.7 are acceptable and are in bold (33). Interpretation of Cronbach's α coefficient (internal consistency): minimally acceptable 0.60–0.64, acceptable 0.65–0.69, good 0.70–0.74, very good 0.75–0.80, and excellent > 0.80 (38). Acceptable values are in bold. Interpretation for sensitivity and specificity: excellent 95–100%, good 85–94.9%, moderate 70–84.9%, and not sensitive or specific <70% (24, 39).CI: confidence interval. Values with acceptable sensitivity and specificity are in bold (> 70%)*.

### Sensitivity and Specificity

When considering all treatments grouped, all items, except the stimuli on the limbs demonstrated sensitivity. On the other hand, only these stimuli demonstrated specificity (Table 7). The sensitivity and specificity of the sum of the scale were 0.96 (CI 0.93–0.99) and 0.83 (CI 0.76–0.89), respectively.

### ROC Curve and Cut-Off Point for Sedation

**Figure 4:**
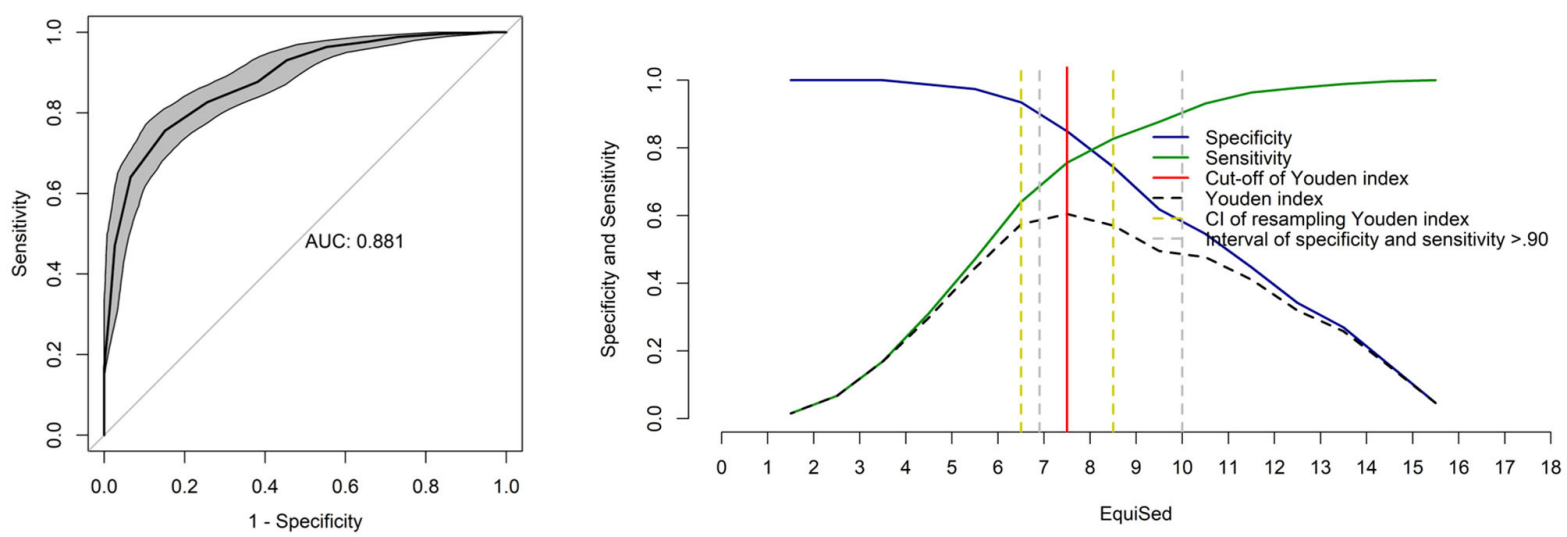
Area under the curve (AUC) and two-graph ROC curve with the diagnostic uncertainty zone for the EquiSed. Receiver operating characteristic (ROC) curve with a 95% confidence interval (CI) calculated from 1,001 replications and under the curve (AUC). Interpretation of AUC: low (0.5–0.7), moderate (0.7–0.9), and high (> 0.9) discriminatory capacity (41). Two-graph ROC curve, CI of 1,001 replications, and sensitivity and specificity >0.90 applied to estimate the diagnostic uncertainty zone of the cut-off point according to the Youden index for the EquiSed. The diagnostic uncertainty zone ranged from 7 to 10; scores <7 are horses that are not sufficiently sedated while scores > 10 are those that are truly sedated. Youden index > 7 represents the cut-off point for sufficient sedation.

The area under the curve was 0.88 and therefore considered to be a moderate discriminatory capacity (Figure 4) (41). The Youden index was > 7 for all pooled evaluators. The range between sensitivity and specificity > 0.90 was between 6.9 and 10. The resampling bootstrap CI was 6.5–8.5. Based on the resampling result, the diagnostic uncertainty zone ranged from 7 to 10, which indicates that scores <7 are horses that are not sufficiently sedated while scores > 10 are those that are truly sedated. The YI were ≥ 5 for NRS, ≥ 1 for SDS and ≥ 43 for VAS.

### Frequency Distribution of Scores

**Figure 5:**
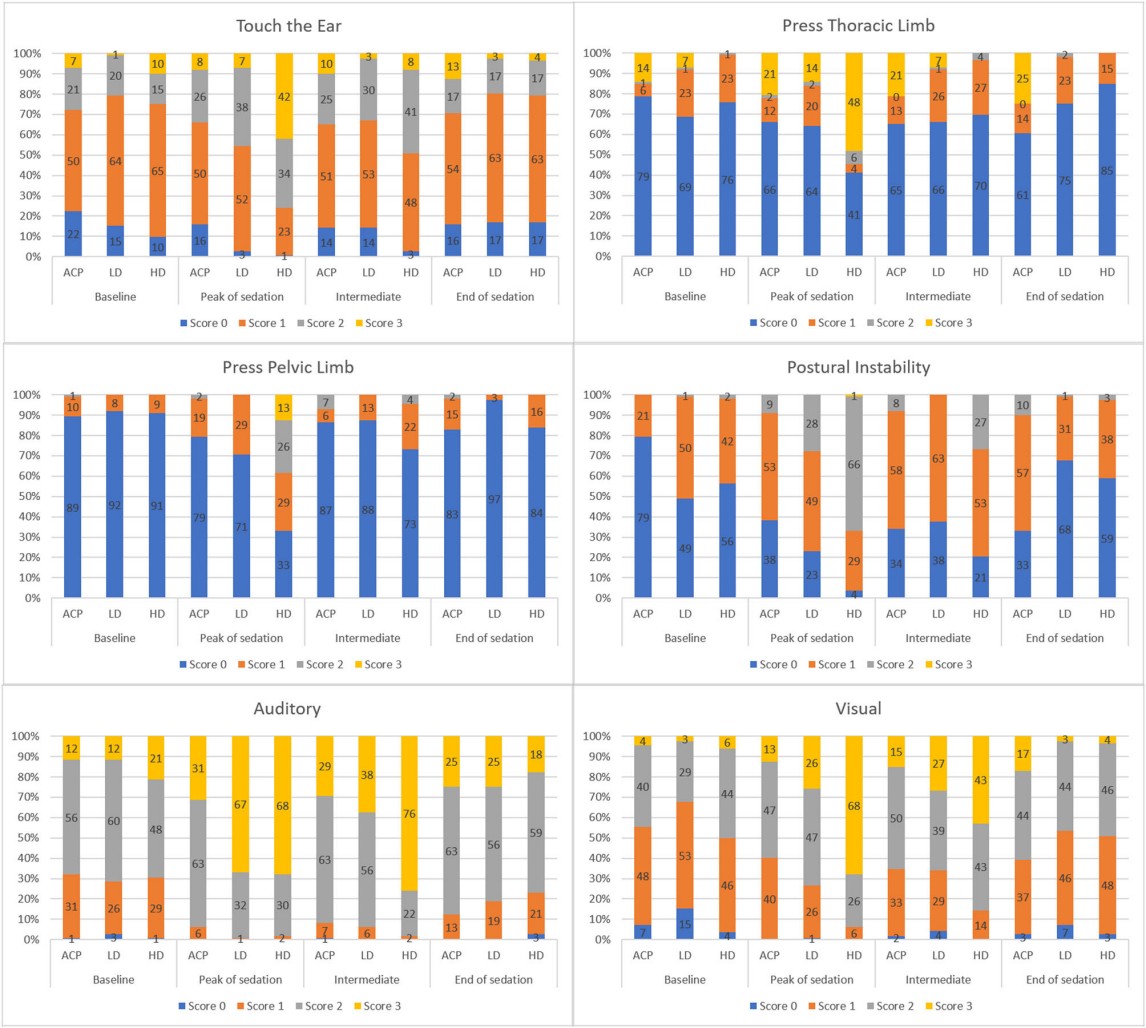
Frequency distribution of EquiSed scores before and after tranquilization (ACP—Treatments ACPL+ACPH), low detomidine (LD—Treatments DL+DLM), and high detomidine (HD—Treatments DH+DHM). ACPL and ACPH—Acepromazine in low and high doses, respectively, DL and DLM—Detomidine in low dose and associated with methadone, respectively, DH and DHM—Detomidine in high dose and associated with methadone, respectively. Baseline—basal sedation, Peak of sedation—peak sedation, Intermediate—intermediate sedation, and End of sedation.

The frequencies of occurrence of the percentage scores for each item of the EquiSed in horses subjected to high detomidine (DH + DHM), low detomidine (DL + DLM), or acepromazine (ACPL + ACPH) are shown in Figure 5.

There was a low frequency of 0 scores for the touch the ears, auditory, and visual stimuli. The frequency of scores of 3 predominated at peak sedation for the auditory stimulus in horses receiving low and high detomidine and for the visual stimulus in horses subjected to high dose detomidine. Although the frequency of scores 3 predominated for pressure on the thoracic limb during peak sedation, there was still a high frequency of scores 0 during high detomidine sedation.

In the stimulus pressure on the pelvic limb, the score 0 predominated at all times. However, for this item, the frequency of higher scores increased proportionally with the intensity of sedation. Postural instability was the item that presented the most consistent results according to the moment and treatment protocols.

## Discussion

The results of this study show that EquiSed differentiated low and deep sedation and is an applicable scale for experimental and clinical studies in horses. Equised identified different levels of sedation according to the validation and reliability criteria used in pain and sedation scales in different animal species and in humans (20–22, 24, 25, 42, 43).

The validation of this scale was based on the observation of the behavioral response of horses first while conscious and without any drugs given, and also after tranquilization/sedation. With the aim to standardize the methodology and guarantee the reproducibility of future studies, the EquiSed compiled the different methods for evaluation of the behavioral responses to the stimuli described since the 1990s (5, 7, 11–13, 15, 19, 31).

Acepromazine and detomidine alone or associated with opioids produce tranquilization and sedation and different degrees of hypo-reactivity to environmental stimuli (7, 18, 30). To validate the EquiSed and to ensure reliability for clinical and experimental use, we selected experienced evaluators familiarized with the use of widely used drugs in equine medicine (2–5, 7, 27, 44, 45). The methodology used for validation and evaluation of intra and interobserver reliability was similar to that described to validate pain scales in animals (20–26), which comprises blinded evaluators and randomized videos to minimize interpretation bias (46). Usually, in other metrics, especially in the area of pain, inexperienced evaluators are included in the validation process, for greater scope in applying the scale (47). Therefore, further validation will be needed when this instrument is used by inexperienced evaluators.

Before the video analysis, content validity was performed. The stimuli already described and reported in the literature were improved and detailed, and its relevance and clarity of the description of all items (35, 48) was assessed by three other experienced researchers in accordance with the process of validation of other animal scales (20–26, 33). Content validity evaluates the representativeness of the items (49) and is correlated with the repeatability and reproducibility of the scale (50).

The four evaluators recruited for our study were experienced veterinary anaesthesiologists. However, general experience does not guarantee good reliability for a specific attribute (51). Reliable scales are essential when interpreting their results (52). Factors that affect reliability include lack of practice/experience, the fatigue of the evaluator, and the inadequate description of the items of a scale (50). Previous studies stated that familiarization with the scale guarantees ability (53, 54), therefore previous training should have ensured good intra- and interobserver reliability in the current study. To reduce fatigue, the video analysis had clear instructions to the evaluators to watch the videos for up to a maximum of 1 h a day.

Despite the previously described cautions, intra- and interobserver reliability for the items auditory and visual stimuli and postural instability presented worse results compared to the other stimulli. These low-reliability values raise the question if these items are reproducible when evaluated in isolation in sedation studies in horses. However, apparently, even the inclusion of these stimuli together with others guaranteed the reliability of the sum of the EquiSed, which presented the highest repeatability of all the scales (NRS, SDS, and VAS); EquiSed had a very good intraobserver reliability and moderate to very good interobserver reliability.

The SDS in this study presented reliabilities ranging from reasonable to very good. These results showed that the reproducibility of the SDS can be compromised when comparing results from different studies. This is mainly due to the fact that unidimensional scales can be subjectively interpreted by the evaluator (2, 3, 5). Similar inconsistent reproducibility results were observed for the SDS when evaluating pain in other species such as dogs (55).

The concurrent criterion validity measures how much the new instrument correlates with a gold standard tool (33, 47). Unfortunately, a validated and therefore gold standard method for sedation assessment in horses has not been available in the literature yet. This did not allow us to have a reliable determination of the accuracy and efficacy of EquiSed. The alternative approach used in the current study for the concurrent criterion validity test was to correlate the EquiSed against unidimensional scales (NRS, VAS, SDS) and HHAG%. This was a similar approach used in previous studies which developed and validated pain scales in other species (20, 26, 56, 57). In addition, HHAG% was incorporated in the current study because this is a well-recognized method for assessing the degree of sedation in horses (2, 5, 7, 11, 12). According to this criteria, EquiSed presented a high correlation with these other scales and these correlations improved as the intensity of sedation increased (acepromazine < low detomidine < high detomidine) (58).

Construct validity evaluates responsiveness. Possible drugs, doses, and associations used in clinical and experimental settings were included to evaluate their effects on the score values. EquiSed identified differences between the moments before and after sedation/tranquilization and differences between treatments and doses. EquiSed scores were proportional to tranquilization/sedation degree, i.e., the highest scores were attributed to the peak and/or intermediate tranquilization/sedation, and the lowest scores were attributed to the baseline. However, the tranquilizing effect of acepromazine overtime remained for up to 120 min (end of sedation) (10, 59). At this time, the effect of acepromazine was even greater than in treatments with detomidine. For this reason, studies using acepromazine should consider its prolonged tranquilizing effect.

The authors recognize that some parameters are not specific for sedation and might be influenced by, for instance, antinociception. But also the opposite, when one investigates the effects of analgesic drugs, may be a confounding factor. In the previously published study performed simultaneously (18), nociceptive threshold was greater and longer in animals treated with the high dose of detomidine and methadone, compared to detomidine alone. Otherwise, the sedation data of the current study showed that the inclusion of methadone did not increase either the total sedation scores or the scores of any separated item. In both previous studies (7, 18), methadone increased detomidine-induced antinociception without increasing sedation. However, even so, it is difficult to differentiate for pelvic and thoracic limb stimuli, sedation from antinociception in horses treated with alpha-2 agonists. None of the sedation scales differentiated the effect of including methadone in each dose of detomidine.

EquiSed detected differences between the high degree of sedation vs. both low degree of sedation and vs. low degree of tranquilization but did not differentiate the high degree of tranquillisation from a low degree of sedation. At the peak of sedation, the total score and the items postural instability and auditory and visual stimulus identified low tranquilization with acepromazine vs. the other groups. Therefore, the scores apparently increased proportionally to the degree of central nervous system (CNS) depression. All items significantly increased the EquiSed score in horses treated with high detomidine, in both peak sedation and/or intermediate sedation. There was very similar responsiveness among the unidimensional scales and EquiSed. As expected, HHAG%, the most used parameter for sedation in horses, likewise the other scales, decreased as detomidine dose increased. If we consider the current interpretation of HHAG% (2), only horses treated with the high detomidine dose could be regarded as sufficiently sedated ( ≤ 50%) in the current study, the value used to define the Youden index (i.e., horses with EquiSed scores > 7 are considered sufficiently sedated).

The PCA identifies how variables are grouped into factors or dimensions (60) to determine the extent of the scale (61). Based on this analysis, all stimuli were approved on the scale (62, 63). The vector analysis showed that all stimuli were directed to the right side of the graph where moments of greater sedation were located, confirming that all the items can identify sedation.

The item-total correlation confirmed the homogeneity and importance of each item on the EquiSed (64), demonstrating that all items contribute to the final score. The internal consistency which correlates the different items on the scale was acceptable for all the items, suggesting that they are representative for evaluating sedation in horses (33).

Sensitivity and specificity evaluate the accuracy in identifying sedated and non-sedated animals, respectively. The α-2 adrenergic agonists reduce the response to different stimuli (7). However, the low sensitivity to tactile stimuli in the limbs in this study may be related to the learning effect already described (5), despite the efforts to minimize this effect by alternating the limb stimulation. Other possible explanations are that the α-2 adrenergic agonist-reduction of tactile stimuli response is dose-dependent. High (20 μg/kg of detomidine) but not low doses reduce the response to tactile stimuli on the limbs (13). Still, some authors sustained that even horses deeply sedated with α-2 adrenergic agonists alone can react to touch (65, 66). The most evident ataxia occurs only from doses > 5 μg/kg (8). In this study, maximum ataxia was not found, which corroborates the low sensitivity to tactile stimuli in the limbs.

With respect to specificity, only the items pressure on the limbs were specific. The lack of specificity of some items is associated with a limitation of the study, where docile horses may not respond to stimulus leading to scores above zero at baseline. It is probable that horses under stress or agitation, and in an unfamiliar environment, unlike our study, would have lowered the scores on all items before sedation as they would respond more readily to stimuli.

The high sensitivity and specificity of the total score of EquiSed compensated for below the expected results of sensitivity and specificity of the isolated items. The HHAG% was used as a predictive value for this calculation because it is an objective and consecrated method to assess sedation (2, 5, 7, 11, 12). Total EquiSed scores >7 (YI) indicates that horses are sufficiently sedated for clinical and or surgical procedures, and this is information may be useful for clinical use.

Although the EquiSed was validated in its entirety with all included items, since the reliability and validity analyses were carried out separately for each item, when, in an experimental or clinical situation, one of the stimuli cannot be used (for example the auditory or visual, as they can frighten animals), they could be excluded, and the scale adapted. However, it is emphasized that a new validation of the adapted scale would be necessary.

Our study is not free of limitations. The first limitation was the docile temperament and previous adaptation of the horses to the environment, personnel, and handling, which possibly explain the lack of specificity of some EquiSed stimuli. To address this deficiency, the scale should be tested in animals with different temperaments to confirm or not this assumption. A second limitation was that the performance of the unidimensional scales might have been inflated by the experience of the evaluators and by prior knowledge of the behavioral indicators of sedation described in the EquiSed. This method of assessment possibly contributed to the very similar responsiveness results among the unidimensional scales and EquiSed. A third limitation is the limited number of horses, however based on the 4 R's (reduce, replace, refine, and respect), the number of horses were reduced to the minimum necessary, but still based on the sample size calculated. The fourth limitation was the lack of independence for observations represented by the replicated use of seven horses submitted to six treatments, evaluated by four observers in 4 moments of two phases, with a total of 168 evaluations. This limitation may lead to an inflated alpha of the hypothesis tests and for the confidence intervals. A final limitation was that 16 videos used for training were selected from the 168 videos used in the main study. Therefore, evaluators could be able to recognize each single horse when assessing the main study videos. However, we do not believe this was a bias because in both cases evaluators were unaware of the moment at which the videos were recorded, and the horses were very similar in appearance.

To summarize, the main advantage of EquiSed over HHAG is the ability to differentiate tranquilization from baseline (without the effect of CNS depressors) and the low level of tranquilization from high level of tranquilization and from low sedation. Besides that, EquiSed, but not HHAG%, was able to differentiate low from high sedation state. The disadvantage of EquiSed is the need for interaction with the horse, especially in clinical practice, contrasting other scales based on facial expression, for example. Otherwise, one of the disadvantages of HHAG is that it cannot be used in dental practice.

EquiSed presents intra- and interobserver reliability and content, criterion, and construct validity in horses tranquilized with acepromazine or sedated with detomidine alone or associated with methadone. EquiSed is easily applicable and differentiates low from high sedation and low from high tranquilization. However, considering that the data replication originated from the grouping of horses, observers and moments may have inflated the alpha values facilitating type I error in results, future experimental and/or clinical studies may either confirm or not the usefulness, reproducibility and validity of the scale.

## Data Availability Statement

The original contributions presented in the study are included in the article/Supplementary Material, further inquiries can be directed to the corresponding author/s.

## Ethics Statement

The animal study was reviewed and approved by Ethics Committee on the Use of Animals for research at the School of Veterinary Medicine and Animal Science, São Paulo State University (Unesp), Botucatu, SP, Brazil, under protocol 2017/0051.

## Author Contributions

ARO contributed to the conception, development, execution, analysis, and interpretation of data and preparation of the manuscript. MG-M contributed to the conception, development, execution, and preparation of the manuscript. MG-M, SKR, and SS contributed to the conception, evaluation of the videos, and corrections of the study manuscript. MWF and JNPPF contributed to the development and execution of the study. PHET contributed with statistical analysis and data interpretation. SPLL contributed to the conception, development, interpretation of data, preparation of the manuscript, and final correction of the manuscript. All authors contributed to the article and approved the submitted version.

## Conflict of Interest

The authors declare that the research was conducted in the absence of any commercial or financial relationships that could be construed as a potential conflict of interest.
