# Supplementary material for: Development, Validation, and Reliability of a Sedation Scale in Horses (EquiSed)
Source: Front Vet Sci. 2021 Feb 16;8:611729. doi: 10.3389/fvets.2021.611729 (PMC7921322; doi:10.3389/fvets.2021.611729)
Supplement: Supplementary file 1 [file Table_1.pdf]

Supplementary Material

Table 1 - Appendix 1. Links to the example videos of each score of EquiSed.

Intensity of sedation			
**Stimuli performed**	**Response to stimuli**	**Scores**	**Links**
**Touch the ear**			
Touch inside the ears with blunt tipped material for three seconds	No response	3	https://youtu.be/NGLSzmW81oc
	Slight movement of the ear and/or head and/or neck	2	https://youtu.be/RFf5QpC1v34
	Intense movement of the ear and/or head and/or neck	1	https://youtu.be/wwcWEu89Tj0
	Intense movement of the ear and head and/or neck and body movement	0	https://youtu.be/agmfgvYPLeM
**Press the coronary band of thoracic limb**			
Apply strong pressure for three seconds with blunt tipped material on the coronary band of thoracic limb	No response	3	https://youtu.be/5Fvx3tMrN8g
	Moves the limb slowly without raising it	2	https://youtu.be/SEZLXEqxNHM
	Raises the limb slowly	1	https://youtu.be/jGBwISkhYe8
	Raises the limb quickly before or when touched and/or moves the other limbs and/or head and/or trunk	0	https://youtu.be/2_ktkYuCONw
**Press the coronary band of pelvic limb**			
Apply strong pressure for three seconds with blunt tipped material on the coronary band of pelvic limb	No response	3	https://youtu.be/14ne5RtGmwM
	Moves the limb slowly without raising it	2	https://youtu.be/THflhScaYvI
	Raises the limb slowly	1	https://youtu.be/pj4nur63x68
	Raises the limb quickly before or when touched and/or moves the other limbs and/or head and/or trunk	0	https://youtu.be/OC4SSO89qiY

Intensity of sedation

Stimuli performed	Response to stimuli	Scores	Links
**Postural instability**			
Observe the stationary animal and then forcefully push it laterally	Intense swaying, risk of falling down or falling down. Abducts (wide stance) the thoracic and/or pelvic limbs, and/or one limb misaligned or crossed	3	https://youtu.be/OQKII9XzHP0
	Moderate swaying. Thoracic and/or pelvic limbs abducted (wide stance), and/or one limb misaligned	2	https://youtu.be/C2NRvdafhLM
	No or slight swaying. One limb abducted (wide stance)	1	https://youtu.be/qARoH38cNS4
	No swaying. Weight bearing on all limbs, or resting one limb	0	https://youtu.be/tmZGMD-wudU
**Auditory**			
Response to loud hand clap behind the animal	No response	3	https://youtu.be/6haTPPUonSw
	Slow movement of the head and/or neck and/or ear(s)	2	https://youtu.be/8wQfxTPfDlE
	Rapid movement of the head and/or neck and/or ear(s)	1	https://youtu.be/4lhm6i9pawY
	Rapid movement of the head and/or neck and/or ear(s) and body movement	0	https://youtu.be/6lCFLiqLit0
**Visual**			
Response to opening an umbrella in front of the animal	No response	3	https://youtu.be/lGpcS2MI7CI
	Slight movement of the head and/or neck and/or ear(s)	2	https://youtu.be/pxN2q3AMdEA
	Intense movement of the head and/or neck and/or ear(s)	1	https://youtu.be/pJ6i9XVub2I
	Moves the head and/or neck and/or ear(s) and limb(s)	0	https://youtu.be/XmtMkjaWuR8
Maximum possible sum of the EquiSed		18	
