# Supplementary material for: Development, Validation, and Reliability of a Sedation Scale in Horses (EquiSed)
Source: Front Vet Sci. 2021 Feb 16;8:611729. doi: 10.3389/fvets.2021.611729 (PMC7921322; doi:10.3389/fvets.2021.611729)
Supplement: Supplementary file 3 [file Data_Sheet_2.pdf]

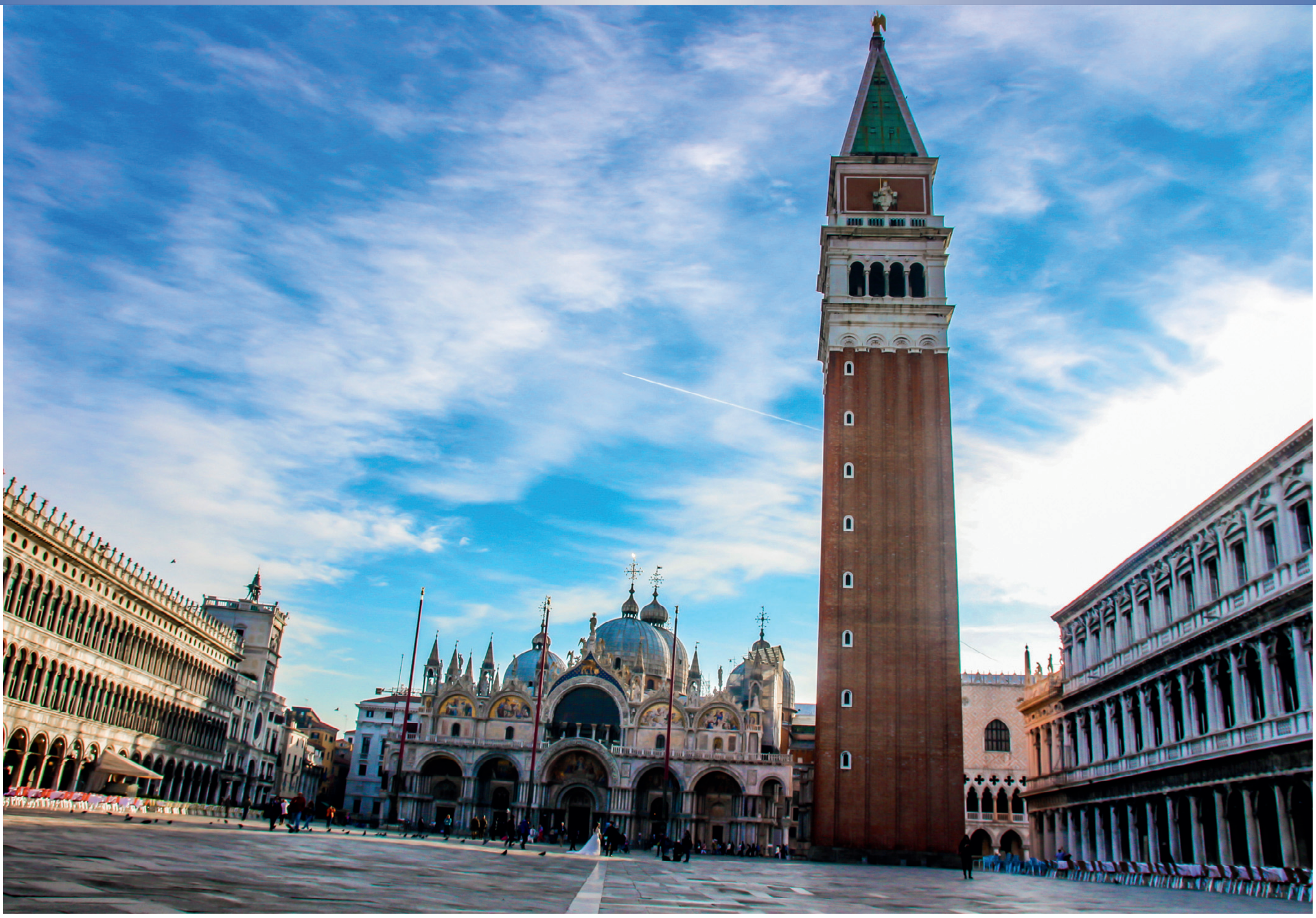

WCVA VENICE 2018

With the support of SIAARTI PRO VITA CONTRA DOLOREM SEMPER

13th WORLD CONGRESS OF VETERINARY ANAESTHESIOLOGY

SEPTEMBER 25TH – 29TH, 2018

PROCEEDINGS

Protective effects of dexmedetomidine-vatinoxan versus dexmedetomidine alone on intestinal ischemia-reperfusion injury in horses under general anaesthesia
Sabine B.R. Kästner 112

Attitude of Brazilian researchers towards pain recognition and control in laboratory animals
Stelio P.L. Luna 112

Anaesthetic effects of fentanyl-lidocaine-ketamine infusion in propofol- or isoflurane-anaesthetized goats undergoing abomasotomy
Eduardo Gutierrez-Blanco 113

The speed of recovery from vecuronium is affected by dose and administration regime
Daniel M. Sakai 113

Postoperative analgesia of an infusion of tramadol or saline in dogs undergoing orthopedic surgery
Marzia Stabile 114

Sedative effects of dexmedetomidine alone or in combination with methadone or midazolam in cats
Mariela Goich 114

Hemodynamics effects of different FiO_2 in calves anesthetized by sevoflurane maintained under pressure-controlled ventilation
Paulo Sergio Patto dos Santos 115

Evaluation of induction and intubation times using fentanyl, ketamine and midazolam in gorillas (Gorilla gorilla gorilla)
Ryan Bailey 115

Perianaesthetic mortality in dogs: preliminary data of a worldwide multicentric study
Jose Ignacio Redondo 116

Perianaesthetic mortality in cats: preliminary data of a worldwide multicentric study
Jose Ignacio Redondo 116

Correlation between central venous and jugular pressures in dorsally recumbent horses under general anesthesia
Eutalio Pimenta 117

Anaesthetic management of dogs with different stages of chronic mitral valve disease in Spain
Filippo Montefiori 117

Is methadone an option for canine gastroduodenoscopy if combined with acepromazine?
Kati Salla 118

Pharmacokinetics, antinociceptive effect and PK/PD modeling of romifidine infusion in standing horses
Sabina Diez Bernal 118

Pharmacokinetics of tramadol following intranasal administration in dogs undergoing ovariohysterectomy
Giorgia della Rocca 119

Effect of cranial hyperflexion of pelvic limbs on interlaminar length of lumbosacral space in sternally and laterally recumbent swine
Chiara Hampton 119

Efficacy of alveolar recruitment in anesthetized horses ventilated with heliox or an oxygen-nitrogen-mixture
Klaus Hopster 120

Effect of levomethadone on the nociceptive withdrawal reflex in horses sedated with romifidine
Nicole Studer 120

Comparison of two anesthetic protocols in food-producing vs. non-food producing wild boars (Sus scrofa)
Johanna Painer 121

Fentanyl causes shivering in isoflurane anaesthetised piglets, dexmedetomidine reduces muscular activity
Henning Andreas Haga 121

Intramuscular alfaxalone-methadone with or without ketamine: effects on sedation and echocardiographic measurements in cats
Eleonora Lazzarini 122

Partial validation of a sedation scale in horses
Alice R. Oliveira 122

Construct and criterion validity, and reliability of the feline grimace scale
Marina Evangelista 123

Analgesic effect of methadone or tramadol on postoperative pain in cats undergoing ovariohysterectomy
Marina Evangelista 123

Analgesic effect of dipyrone or meloxicam on postoperative pain in cats undergoing ovariohysterectomy
Marina Evangelista 124

ID-172 Intramuscular alfaxalone-methadone with or without ketamine: effects on sedation and echocardiographic measurements in cats

○ Eleonora Lazzarini, DVM[1], Elisa Martinelli, DVM, PhD[1], Federica A. Brioschi, DVM[1], Daniela Gioeni, DVM[1], Roberto Toschi Corneliani, DVM[1], Alessandra Carotenuto, DVM[1]
[1] Ospedale Veterinario San Francesco, Milano, Italy

The aim of this study was to evaluate the effect of IM alfaxalone-methadone, with or without ketamine, on sedation and echocardiographic measurements in healthy cats.

Twenty-two client-owned cats undergoing neutering were enrolled. All cats underwent two standard echocardiographic evaluations: at baseline (bECG) and under sedation (sECG). Cats randomly received IM alfaxalone (2 mg kg^{-1}) and methadone (0.3 mg kg^{-1}) with (AMK group, n = 11) or without (AM group, n = 11) ketamine (1 mg kg^{-1}). The sedation level (Biermann et al. 2012) from 0 (no sedation) to 5 (good quality of sedation) was assessed at 5 (T5), 10 (T10) and 15 (T15) minutes after IM injection. Values from bECG and sECG (performed at T15) were compared. Data were analysed using the Student's t-test and the Mann-Whitney U-test.

Twenty-one cats were included and considered sufficient to have a power of 80% and a type I error of 5%. Sedation score was significantly higher in AMK (n = 11) than in AM group (n = 10) at any observation time (Tab.1). Values from bECG and sECG showed no significant differences with exception for ejection time in AM group (p = 0.03) and pulmonary peak velocity in AMK group (p = 0.02).

In healthy cats both protocols had no meaningful effects on the echocardiographic variables. The AM combination led to poor sedative effects, however the addition of ketamine improves the sedation level.

In accordance with the Italian law an informed written consent was obtained from the owner so as to collect data for this study, which was consistent with neutering procedure.

Table 1 - Sedation score

	T5	T10	T15
AM	0.5 (0-4)	1.5 (0-5)	2 (0-5)
AMK	4 (1-5)	4 (1-5)	4 (1-5)

References

Biermann K, Hugerbühler S, Mischke R et al. (2012) Sedative, cardiovascular, haematologic and biochemical effects of four different drug combinations administered intramuscularly in cats. Vet Anaesth Analg 39, 137-150.

ID-174 Partial validation of a sedation scale in horses

○ Alice R. Oliveira, DVM, MSc[1], Miguel Gozalo-Marcilla, DVM, MSc, PhD, Dip ECVAA[2], Mariana W. Fonseca, DVM[2], José N.P. Puoli Filho, DVM, MSc, Phd[1], Stelio P.L. Luna, DVM, MSc, PhD, Dip ECVAA, IVAS certified[1]
[1] FMVZ-Unesp, Botucatu, Brazil
[2] FMB-Unesp, Botucatu, Brazil

This crossover study aimed to validate a sedation scale in horses.

Seven horses were randomly allocated to IV weekly treatment with saline (SAL), acepromazine (GA) (0.09 mg kg^{-1}), detomidine (GD) (5 $\mu g\ kg^{-1}$ + 12.5 $\mu g\ kg^{-1}$ hour) and detomidine combined with methadone (GDM) (0.2 mg kg^{-1} + 0.05 mg kg^{-1} hour). Ataxia and response to tactile stimuli to the ears, hindlimb, and forelimb coronary bands, auditory and visual stimuli were each scored from zero to three, and the scores added. Height of head above the ground (HHAG) was measured. Responsiveness was evaluated by Friedman´s test, relevance of each score by odds ratio and Spearman`s test was used to correlate sedation scale *versus* VAS and HHAG.

Maximum sedation in GD, GDM, and GA was 39, 50 and 27% greater than baseline respectively ($p < 0.05$). There was strong correlation between sedation scale and VAS (r = 0.68, $p < 0.0001$) and moderate and negative correlation with HHAG (r= -0.48, $p < 0.0001$). The specificity was excellent for tactille (< 4.9%), and good for auditory (13%) and visual (8.3%) stimuli. Results from odds ratio showed that compared to SAL, the chances to present high sedation scores (2 and 3) were greater in GD and GDM and chances to present high ataxia scores were greater in GDM. We concluded the scale showed responsiveness, specificity and was able to detect sedation produced by an α_2 agonist alone or combined with opioid.

Ethical Statement: this study was approved by the Institutional Ethical Committee (0051/2017).

Owner consent: All animals belonged to the Institutional farm.

Acknowledgments: São Paulo Research Foundation (FAPESP) for funding support (grant 2017/16208-0 and thematic project 2017/12815-0).
