# Supplementary material for: Development, Validation, and Reliability of a Sedation Scale in Horses (EquiSed)
Source: Front Vet Sci. 2021 Feb 16;8:611729. doi: 10.3389/fvets.2021.611729 (PMC7921322; doi:10.3389/fvets.2021.611729)
Supplement: Supplementary file 4 [file Data_Sheet_3.pdf]

AVA AUTUMN MEETING 2019
PROGRAMME BOOK

AVA

Association of Veterinary Anaesthetists

11-13 September 2019

NH Hotel Belfort

Ghent - Belgium

2019

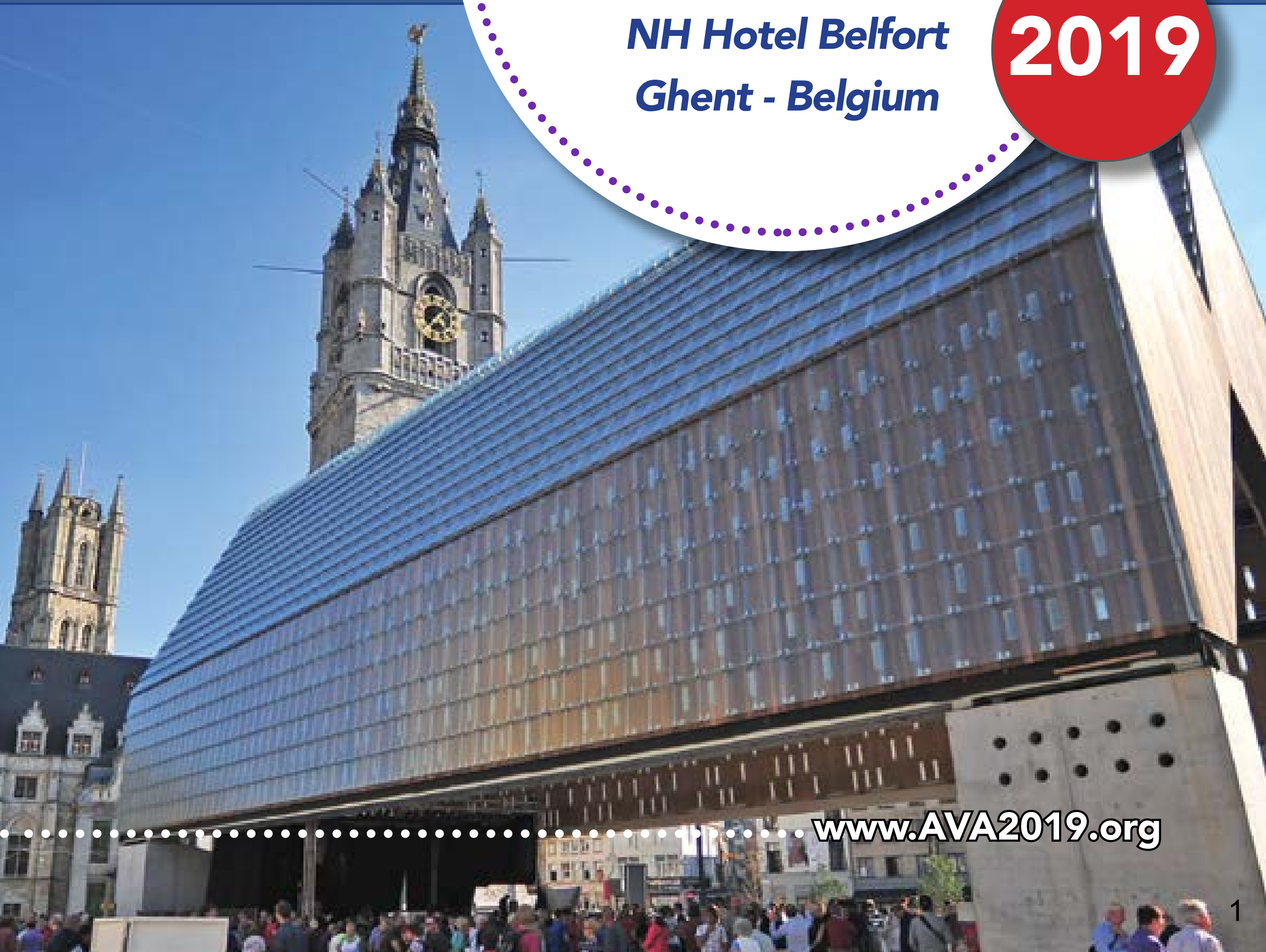

www.AVA2019.org

Reliability and odds ratio of different evaluators in assessing sedation in horses

Oliveira AR, Gozalo-Marcilla M, Schauvliege S, Ringer SK, Puoli-Filho JNP, Luna SPL.

Department of Veterinary Surgery and Anaesthesiology, School of Veterinary Medicine and Animal Science (FMVZ); São Paulo State University (Unesp), Botucatu, SP, Brazil
The Royal (Dick) School of Veterinary Studies and the Roslin Institute, The University of Edinburgh, Easter Bush Campus, Midlothian, UK
Department of Surgery and Anaesthesia of Domestic Animal, Faculty of Veterinary Medicine, Ghent University, Merelbeke, Belgium
Equine Department, Anaesthesiology Section, Vetsuisse Faculty, University of Zurich, Zurich, Switzerland
Department of Animal Production, School of Veterinary Medicine and Animal Science (FMVZ); São Paulo State University (Unesp), Botucatu, SP, Brazil

Reproducibility is an indispensable test to guarantee validation of a measurement and previous training is an essential step to ensure reliability. This study aimed to evaluate intra and inter-reliability and odds ratio of four observers in testing two scales for evaluation of sedation in horses.
Four experienced anaesthesiologists scored a facial (photos) and a behavioural sedation scale (videos of 45 seconds) of horses submitted to different sedation protocols. The digital media, including unsedated and slightly and deeply sedated horses, was evaluated twice, with a three weeks interval. Inter and intra-reliability were calculated by Spearman correlation. The chances of each evaluator identifying sedation using the facial scale were calculated by odds ratio.
Inter-reliability ranged between 79 and 92% for the facial and from 84 to 89% for the behavioural scale. Intra-reliability for the facial scale was 95%, 87%, 95% and 94% and for the behavioural scale was 88%, 82%, 91% and 85% for evaluator 1 (E1), E2, E3 and E4 respectively.
Table 1: Odds ratio of facial changes to differentiate sedated *versus* unsedated horses.

	**Ears**	**Eyes**	**Lower lip**	**Upper lip**
**E1**	85.00*	44.20*	85.00*	21.00*
**E2**	10.82	10.82	26.71*	10.82
**E3**	6.53	10.82	6.53	26.71*
**E4**	17	17	44.20*	44.20*

* $p < 0.0001$
All observers showed excellent intra and inter-reliability, indicating that these evaluators may be selected for a validation process. Odds ratio varied among the observers and according to the different items of the facial scale, showing that E1 was the best evaluator.
